# Complication Rates After Ultrasonography-Guided Nerve Blocks Performed in the Emergency Department

**DOI:** 10.1001/jamanetworkopen.2024.44742

**Published:** 2024-11-13

**Authors:** Andrew Goldsmith, Lachlan Driver, Nicole M. Duggan, Matthew Riscinti, David Martin, Michael Heffler, Hamid Shokoohi, Andrea Dreyfuss, Jordan Sell, Calvin Brown, Christopher Fung, Leland Perice, Daniel Bennett, Natalie Truong, S. Zan Jafry, Michael Macias, Joseph Brown, Arun Nagdev

**Affiliations:** 1 Department of Emergency Medicine, Lahey Hospital and Medical Center, University of Massachusetts Chan Medical School, Burlington; 2 Department of Emergency Medicine, Brigham and Women’s Hospital, Harvard Medical School, Boston, Massachusetts; 3 Department of Emergency Medicine, Massachusetts General Hospital, Harvard Medical School, Boston; 4 Department of Emergency Medicine, Denver Health and Hospital Authority, University of Colorado School of Medicine, Denver; 5 Department of Emergency Medicine, Wilma Chan Highland Hospital Campus, Alameda Health System, University of California, San Francisco; 6 Department of Emergency Medicine, University of Colorado Anschutz Medical Campus, Aurora; 7 Department of Emergency Medicine, Hennepin Healthcare, Minneapolis, Minnesota; 8 Department of Emergency Medicine, University of Michigan Medical School, Ann Arbor; 9 Department of Emergency Medicine, The Warren Alpert Medical School, Brown University, Providence, Rhode Island; 10 Department of Emergency Medicine, Loma Linda University, Loma Linda, California; 11 Department of Emergency Medicine, Temecula Valley Hospital, Temecula, California

## Abstract

**Importance:**

Ultrasonography-guided nerve blocks (UGNBs) have become a core component of multimodal analgesia for acute pain management in the emergency department (ED). Despite their growing use, national adoption of UGNBs has been slow due to a lack of procedural safety in the ED.

**Objective:**

To assess the complication rates and patient pain scores of UGNBs performed in the ED.

**Design, Setting, and Participants:**

This cohort study included data from the National Ultrasound-Guided Nerve Block Registry, a retrospective multicenter observational registry encompassing procedures performed in 11 EDs in the US from January 1, 2022, to December 31, 2023, of adult patients who underwent a UGNB.

**Exposure:**

UGNB encounters.

**Main Outcomes and Measures:**

The primary outcome of this study was complication rates associated with ED-performed UGNBs recorded in the National Ultrasound-Guided Nerve Block Registry from January 1, 2022, to December 31, 2023. The secondary outcome was patient pain scores of ED-based UGNBs. Data for all adult patients who underwent an ED-based UGNB at each site were recorded. The volume of UGNB at each site, as well as procedural outcomes (including complications), were recorded. Data were analyzed using descriptive statistics of all variables.

**Results:**

In total, 2735 UGNB encounters among adult patients (median age, 62 years [IQR, 41-77 years]; 51.6% male) across 11 EDs nationwide were analyzed. Fascia iliaca blocks were the most commonly performed UGNBs (975 of 2742 blocks [35.6%]). Complications occurred at a rate of 0.4% (10 of 2735 blocks). One episode of local anesthetic systemic toxicity requiring an intralipid was reported. Overall, 1320 of 1864 patients (70.8%) experienced 51% to 100% pain relief following UGNBs. Operator training level varied, although 1953 of 2733 procedures (71.5%) were performed by resident physicians.

**Conclusions and Relevance:**

The findings of this cohort study of 2735 UGNB encounters support the safety of UGNBs in ED settings and suggest an association with improvement in patient pain scores. Broader implementation of UGNBs in ED settings may have important implications as key elements of multimodal analgesia strategies to reduce opioid use and improve patient care.

## Key Points

**Question:**

Are ultrasonography-guided nerve blocks (UGNBs) performed in the emergency department safe?

**Findings:**

In this cohort study of 2742 UGNBs performed across 11 emergency departments, patients experienced a complication rate of 0.4%. One major complication was reported, with no long-term adverse outcomes.

**Meaning:**

These findings suggest that UGNBs may be safe when performed by emergency clinicians, offering a promising avenue for multimodal analgesia strategies.

## Teaser

This cohort study uses data from a US registry to assess the complication rates of ultrasonography-guided nerve blocks performed in an emergency department (ED) setting as well as the associated improvements in patient pain scores.

## Introduction

Ultrasonography-guided nerve blocks (UGNBs) are a critical component of multimodal analgesia across medical specialties. Along with offering optimal analgesia and reducing the reliance on opioids, these procedures can reduce health care–associated complications.^1,2,3,4,5,6,7^ UGNBs have become a staple of both perioperative pain control and chronic pain management in outpatient clinics. Currently, pain medicine fellowships that emphasize training in UGNBs are attracting applicants from more than 20 different graduate medical education fields, thus emphasizing the multidisciplinary benefits of pain management education and training.^8,9^

The short-term and long-term deleterious effects of opioid use (tolerance, misuse, opioid-induced hyperalgesia, physical dependence, falls, and addiction) are broadly documented.^10,11,12,13,14,15,16,17,18^ Unfortunately, given the often inadequate pain control achieved with medications such as nonsteroidal anti-inflammatory drugs and acetaminophen for acutely painful conditions, opioid use has become commonplace in the emergency department (ED) setting. Along with the deleterious effects, use of opioids in the ED has been shown to increase health care costs and lead to poor patient outcomes.^16,17,18,19,20,21^ As such, ED-based pain management is beginning to rely on multimodal approaches to maximize pain management, minimize complications, and improve patient care.

Over the past decade, numerous case series, retrospective studies, and a few trials on specific ED-performed nerve blocks have suggested that UGNBs performed by ED clinicians may be safe and potentially effective for various acute injuries.^22,23,24^ However, a large study encompassing all ED UGNBs across multiple EDs has never been evaluated. A recent American College of Emergency Physicians’ policy statement delineated UGNBs as not only falling within the scope of ED-based practice but also named them as a critical component of ED-based pain management.^25,26^ Other specialties now also recognize UGNBs as a cornerstone of ED-based care including the American Academy of Orthopaedic Surgeons and the American College of Surgeons, which in 2020 published best practices guidelines endorsing UGNBs as a standard of care for pain management in patients presenting with traumatic injuries.^25,26,27,28^

While some EDs have begun to embrace the use of UGNBs, wide-scale adoption is lacking. Barriers to widespread use include lack of procedural training and competency, unclear incidence of complications (eg, peripheral nerve injury, local anesthetic systemic toxicity), and uncertainty surrounding procedural efficacy when performed in the ED.^29^ Current safety and efficacy data have been limited to single institutions with variable reporting and low sample sizes.^30^ To our knowledge, there have been no large-scale multicenter analyses defining the safety and efficacy of UGNBs performed by emergency clinicians in the US.

Our study presents the findings from the National Ultrasound-Guided Nerve (NURVE) Block Registry, a multicenter clinical registry developed by 3 authors (A.G., J.B., and A.N.) with contributions from all authors. To our knowledge, the registry is the first to assess procedural practice and outcomes of ED-performed UGNBs. Our overall aim was to define both safety and efficacy profiles of UGNBs being performed by ED physicians for acute painful conditions across the US.

## Methods

### Study Design and Setting

The NURVE Block Registry is a multicenter retrospective registry of UGNBs performed in 11 EDs in the US from January 1, 2022, to December 31, 2023. Sites self-selected for participation in the registry. The institutional review board of each participating site approved this cohort study prior to data collection and analysis and waived the need for informed consent owing to the use of deidentified data. This study was performed in adherence with the Strengthening the Reporting of Observational Studies in Epidemiology (STROBE) reporting guideline.

### Selection of Patients

All adult patients 18 years or older who underwent a UGNB in participating EDs during the study dates were included. Nerve blocks without ultrasonography guidance were excluded. To ensure the sufficient capture of UGNBs and that the dataset was representative of national practice patterns, each participating site director was required to confirm that greater than 90% of UGNBs were reported to the registry from each site to contribute data to the NURVE Block Registry’s database. In this dataset, all sites recorded 100% of their queried electronic medical record (EMR) UGNB procedure notes. This was queried against all procedure-documented templates to ensure that we were over 90% compliant.

### Data Collection and Management

Each site’s director was responsible for entering all registry data. Each site used a mixture of trained physicians and/or research assistants to complete the medical record review. Nonphysicians received at least 1 hour of training prior to the medical record review. Medical records were located by an EMR query of all UGNB procedure notes completed within the ED. All data were collected by retrospective medical record review and were inputted into a standardized data collection form designed by lead study staff (A.G., L.D., J.B., and A.N.) and distributed among participating sites. A subset of data at each site was reviewed for accuracy, and if a disagreement occurred, a third reviewer served as a tiebreaker. Participating sites queried and submitted data twice yearly to the coordinating center (Brigham and Women’s Hospital) for compliance. A study staff member at the coordinating center (L.D.) reviewed reports for compliance.

Data on delayed complications, such as peripheral nerve injury (PNI), were collected by reviewing the medical record 30 days after the procedure through the local EMR and, if possible, an interoperability platform. The research team at each site entered collected site data into a web-based data collection from REDCap for each UGNB.^31,32^ Each site’s data were transferred to the coordinating center for quality assurance and analysis.

### Outcome Measures

The primary outcome of this study was to assess the complication rate associated with ED-based UGNBs recorded in the NURVE Block Registry. The secondary outcome was to assess the associated patient pain scores of ED-based UGNBs. Missing data for specific patient variables (ie, pain scores) are presented, given the limitation of retrospective review.

Patient variables collected included demographic data, with identification of the patient’s sex, race, and ethnicity based on the EMR report, and body mass index. Race categories included American Indian or Alaska Native, Asian or Pacific Islander, Black or African American, White, and other (as documented in the EMR and not reflective of specific subcategories). Ethnicity categories included Hispanic or Latinx and non-Hispanic or non-Latinx. Race and ethnicity were ascertained by self-report by individual hospital reporting policies in the EMR; these data were collected because they are self-reported per individual hospital reporting policies in the EHR. Procedural data collected included the specific type of UGNB performed, the clinical indication for UGNB, the type of anesthetic used, the type of adjunct medication used, and the level of training of the proceduralist. Outcome data collected included preprocedure and postprocedure visual analog scale pain scores, as well as both immediate and delayed and major and minor procedural complications. Immediate procedural complications were defined as any changes in hemodynamic status, compartment syndrome, and/or injuries directly resulting from the UGNB (eg, falls). The immediate time period was defined as any complication that occurred while the patient was in the ED during their initial encounter. Delayed complications (eg, PNI) were any complications identified after ED disposition from the date of service up to 30 days after the patient received the UGNB.

Major complications were defined a priori as local anesthetic toxicity syndrome, PNI, pneumothorax, and/or a delay in diagnosis of compartment syndrome. Minor complications were defined as any musculoskeletal injuries resulting from temporary muscle weakness or sensory loss in extremities; 2 of the authors (A.G. and A.N.) determined the type of complication as referred by the individual site. If there was a disagreement, 1 author (J.B.) provided the deciding vote. Three authors (A.G., A.N., and J.B.) have over 30 years of combined experience performing UGNBs in EDs.

Pain scores were measured using a visual analog scale, ranging from 0 to 10, with 0 being no pain and 10 being the most severe pain. The percent change in UGNB-related pain was recorded from before to after the block at the time of disposition and was grouped into quartiles (eg, 0%-25%, 26%-50%). These scores were recorded in the EMR by either registered nurses or by clinicians, depending on the institution. If there was a discrepancy in the postblock scores, the smaller improvement in pain score was used.

### Statistical Analysis

Data were exported from REDCap to Microsoft Excel (Microsoft Corp) for statistical analyses. Descriptive statistics were performed. Continuous variables were presented as medians with IQRs, while categorical variables were presented as frequencies and percentages.

Raw agreement was used to evaluate interrater reliability. The mean (SD) raw agreement across all 11 sites was 0.95 (0.05). To calculate raw agreement, a second independent reviewer analyzed data from 10 randomly selected patients at each of the 11 ED sites, recording the proportion of cases in which the reviewer’s assessment matched the initial assessment across 25 variables per patient. The raw agreement rate for each site was then averaged to obtain an overall measure of agreement. Two-sided *P* < .05 was considered statistically significant.

## Results

### Patient and Enrolling Site Demographics

In total, 2735 UGNB patient encounters across 11 EDs nationwide were analyzed. Among patients, the median age was 62 years (IQR, 41-77 years), 1316 of 2727 (48.3%) were female and 1406 of 2727 (51.6%) were male. Of the 2484 patients with data on race, most UGNBs were performed on White patients (1833 [73.8%]), followed by those who were Black or African American (409 [16.5%]). Among the other race categories, 31 (1.2%) UGNBs were performed on patients who were American Indian or Alaska Native, 181 (7.3%) Asian or Pacific Islander, and 30 (1.2%) other race. Of the 2654 ethnicity categories with data, UGNBs were performed on 533 (20.1%) Hispanic or Latinx patients and 2121 (79.9%) non-Hispanic or non-Latinx patients (Table 1). Of the 11 national sites, 2 were considered community EDs, and 9 had academic affiliations with residency training programs. Eight of the 11 sites had associated advanced emergency ultrasonography fellowships. The range of ED census across the 11 sites was 35 000 to 130 000 patients (eTable in Supplement 1). Of the 11 hospitals, each had at least 1 ultrasonography fellowship–trained physician. All academic sites were level 1 trauma centers, while the community sites were not trauma centers. Ten of 11 sites were considered to be in urban geographic areas. The highest percentage of blocks performed by an ultrasonography fellowship–trained attending physician was at site number 10, which included a total of 28.1% (81 of 288).

**Table 1.** Summary Statistics for the NURVE Block Registry

Characteristic	Participants (N = 2735)^a^
Age, median (IQR), y^b^	62 (41-77)
Weight, median (IQR), kg^b^	73.7 (62.0-86.2)
Height, median (IQR), cm^b^	167.6 (157.5-177.8)
BMI, median (IQR)^b^	22.6 (22.7-25.8)
Encounters with blocks per ED site, median (IQR)	157 (129-278)
Sex	
Total patients with data	2727 (99.7)
Female	1316 (48.3)
Male	1406 (51.6)
Nonbinary or other gender	5 (0.2)
Race	
Total patients with data	2484 (90.8)
American Indian or Alaska Native	31 (1.2)
Asian or Pacific Islander	181 (7.3)
Black or African American	409 (16.5)
White	1833 (73.8)
Other^c^	30 (1.2)
Ethnicity	
Total patients with data	2654 (97.0)
Hispanic or Latinx	533 (20.1)
Non-Hispanic or non-Latinx	2121 (79.9)
Patient use of antiplatelet or anticoagulation	
Total patients with data	2179 (80.0)
None	1888 (86.6)
ASA	117 (5.3)
DOAC or NOAC	116 (5.3)
Warfarin	19 (0.9)
Enoxaparin	2 (0.0)
Antiplatelet (eg, clopidogrel, ticagrelor)	37 (1.7)
Operator training level	
Total patients with data	2733 (99.9)
Attending (nonultrasonography fellowship– trained)	221 (8.1)
Ultrasonography fellow	139 (5.1)
Attending (ultrasonography fellowship–trained)	244 (8.9)
APP	176 (6.4)
EM resident or intern	1953 (71.5)
Blocks that operator has previously performed, No.	
Total patients with data	2198 (80.4)
0-5	430 (19.6)
6-10	366 (16.7)
11-20	512 (23.3)
>20	890 (40.5)

Abbreviations: APP, advanced practice practitioners (ie, physician assistant or nurse practitioner); ASA, acetylsalicylic acid (aspirin); BMI, body mass index (calculated as weight in kilograms divided by height in meters squared); DOAC or NOAC, direct oral anticoagulant or novel oral anticoagulant (eg, apixaban); ED, emergency department; EM, emergency medicine; NURVE, National Ultrasound-Guided Nerve.

^a^ Data are presented as No. (%) unless otherwise indicated.

^b^ The unknown value for age is 5, weight is 315, height is 452, and BMI is 479.

^c^ Other race was documented in the electronic medical record and was not reflective of specific subcategories.

### Additional Patient Demographic Information

**Figure 1.**
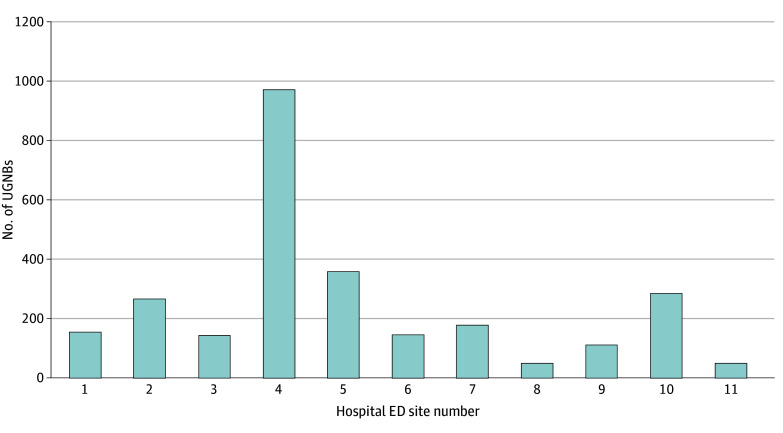
Distribution of the Total Number of Encounters (N = 2735) With Ultrasonography-Guided Nerve Blocks (UGNBs) at 11 Hospital Emergency Department (ED) Sites Hospital ED sites that were a part of the registry were randomly assigned a site number 1 through 11.

In total, 2735 encounters with 2742 total UGNBs were documented across 11 national sites as a part of the NURVE Block Registry. The median number of patients who underwent nerve blocks per site was 157 (IQR, 129-278) (Figure 1). Overall, of the available data for 2560 UGNBs, 2296 (89.7%) were used for pain control, 246 (9.6%) were used for procedural analgesia, and 18 (0.7%) were used for both.

Among the patients undergoing UGNBs, the median weight was 73.7 kg (IQR, 62.0-86.2 kg), height was 167.6 cm (IQR, 157.5-177.8 cm), and body mass index was 22.6 (IQR, 22.7-25.8), calculated as weight in kilograms divided by height in meters squared. Direct oral anticoagulants and/or warfarin were actively used by 135 of 2179 patients (6.2%) at the time of enrollment in the registry (Table 1).

### Operator Experience and Training

The operator training level predominantly included residents (1953 of 2733 [71.5%]) with attending supervision. There were 221 of 2733 attending physicians (8.1%) with no ultrasonography fellowship training represented of the UGNBs performed. The experience level of the operator varied in terms of volume of UGNBs performed. Operators with over 20 prior UGNBs performed represented 890 of 2198 (40.5%) of UGNBs in the registry, while operators with 5 or fewer total prior UGNBs performed represented 430 of 2198 (19.6%) of the procedures (Table 1).

### UGNB Types and Indications

**Figure 2.**
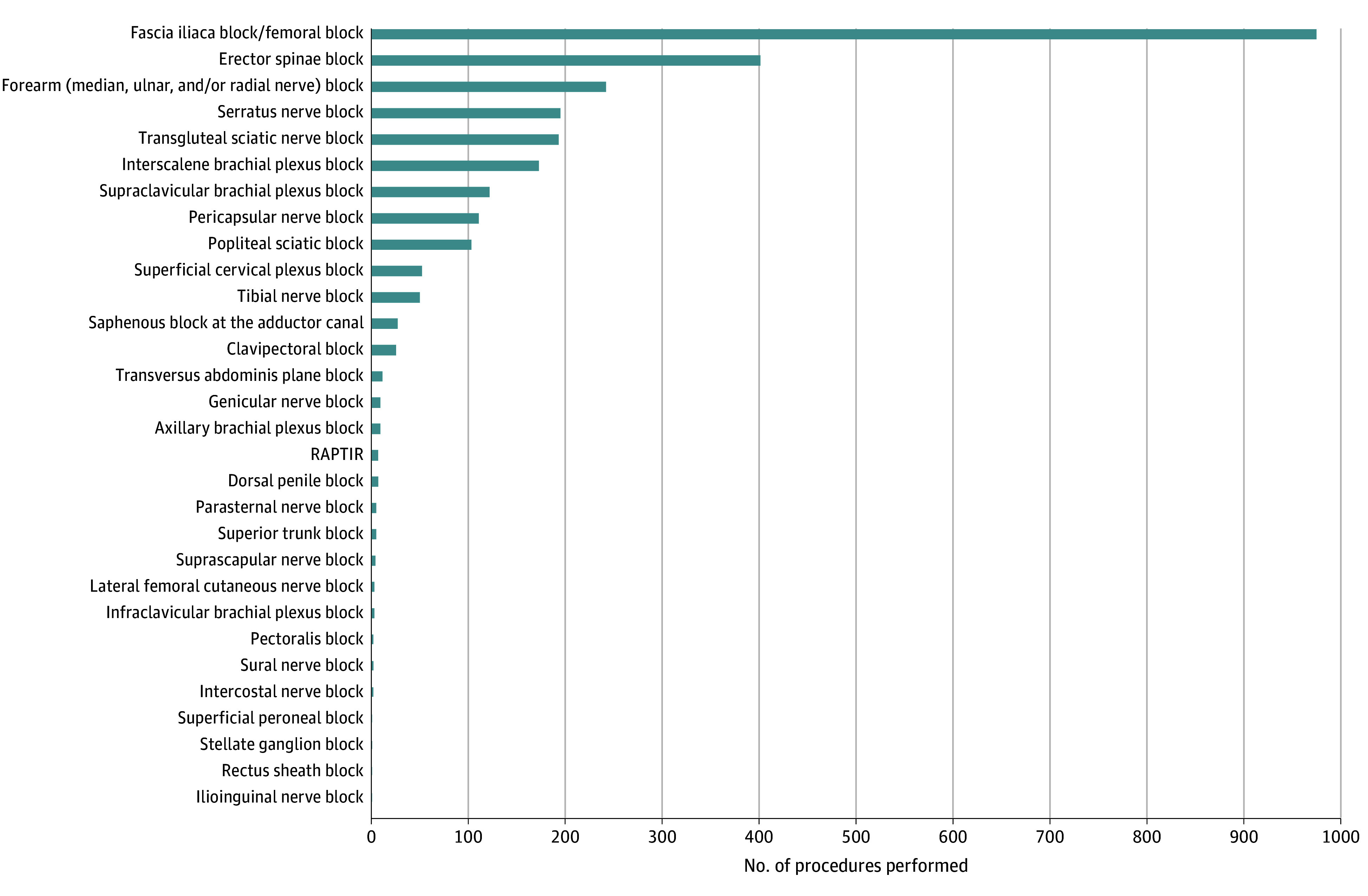
Frequency of Nerve Block Procedure Types Performed in the Study The total number of blocks in the registry was 2742, with 7 patients receiving 2 blocks each. RAPTIR indicates retroclavicular approach to the infraclavicular region.

Among the 2742 UGNB types, the most commonly performed were the fascia iliaca block or femoral nerve block (975 [35.6%]), erector spinae plane block (401 [14.6%]), and forearm (eg, median, ulnar, and/or radial nerve) blocks (242 [8.8%]). Less common procedures, such as the stellate ganglion block and rectus sheath block, were each only performed once (Figure 2).

### UGNB Complication Rates

Among 2735 encounters, 1 major complication was reported in the primary outcome, resulting in a rate of 0.04%. This complication involved a case of local anesthetic toxicity requiring intralipid administration. Additionally, 9 minor complications were reported, accounting for 9 (0.33%) of the UGNB procedures. These minor complications included hoarseness, breathing difficulty, hypotension, and transient nerve-related symptoms like numbness and weakness. Among the 10 encounters with complications, 6 (60%) required admission and/or ED observation unit admission, while 3 (30%) were discharged home. Notably, 9 (90%) of the complications were immediate, with 1 (10%) reported as delayed. Further details of each complication are outlined in Table 2.

**Table 2.** All Complications in the NURVE Block Registry and Related Procedure Details

Case number	Nerve block	Complication	Type	Anesthetic, % (unit of measure)	Disposition	Procedure or complication details
28	Supraclavicular brachial plexus	Hoarseness	Minor, immediate	Ropivacaine, 0.5 (25 mL)	Discharged	The patient experienced hoarseness, which was thought to be due to a unilateral laryngeal nerve blockade.
92	Superficial cervical plexus	Hoarseness	Minor, immediate	Lidocaine, 1 (12 mL)	Inpatient	The patient experienced hoarseness, which was thought to be due to a unilateral laryngeal nerve blockade.
138	TGSNB	Numbness or weakness	Minor, immediate	Ropivacaine, 0.5 (15 mL)	Discharged	The patient had difficulty walking after the procedure secondary to a foot drop; was discharged home with crutches; and pain continued to improve while home.
142	Erector spinae plane	LAST	Major, immediate	Ropivacaine, 1 (20 mL)	Inpatient	The patient endorsed anxiety and high blood pressure, and their symptoms initially improved with an intralipid. However, when they were receiving a second dose of an intralipid, they became more anxious and requested cessation of treatment. Per clinicians’ assessment, the suspicion of a true case of LAST was low.
214	TGSNB	Breathing difficulty	Minor, immediate	Bupivacaine, 0.5 (10 mL)	ED observation	The patient was placed in ED observation due to an adverse reaction after the block, which included symptoms such as hyperventilation, anxiety, and amnesia.
256	Fascia iliaca or femoral	Hypotension	Minor, immediate	Bupivacaine, 0.5 (30 mL) + dexamethasone (10 mg)	Inpatient	After the procedure, the patient became hypotensive and somnolent. It was initially thought that they might be bleeding from a fall, and a large left proximal thigh hematoma was found without active contrast extravasation. The patient’s condition may be attributed to a vagal response or to the hematoma from the fall, although this is unclear.
349	TGSNB	Numbness or weakness	Minor, delayed	Bupivacaine, 0.5 (10 mL) + dexamethasone (10 mg)	Discharged	After leaving, the patient sprained their ankle and had to be admitted to observation on the same day.
529	TGSNB	Severe pain at nerve distribution	Minor, immediate	Bupivacaine, 0.25 (30 mL) + dexamethasone (10 mg)	ED observation	The patient experienced severe nerve pain starting in their lower back and shooting up their spine. They had no specific midline spinal tenderness but exhibited diffuse left paraspinal tenderness.
596	TGSNB	Numbness or weakness	Minor, immediate	Bupivacaine 0.5 (30 mL) + dexamethasone (10 mg)	Inpatient	After receiving a nerve block, the patient experienced numbness from the lateral shin down to the foot and developed a temporary foot drop. An MRI revealed a malignancy at the L4 level.
983	Supraclavicular brachial plexus	Hoarseness	Minor, immediate	Ropivacaine, 0.5 (30 mL)	Inpatient	The patient experienced hemidiaphragmatic paralysis, which led to the need for supplemental oxygen and hoarseness. They also developed Horner syndrome.

Abbreviations: ED, emergency department; LAST, local anesthetic systemic toxicity; MRI, magnetic resonance imaging; NURVE, National Ultrasound-Guided Nerve; TGSNB, transgluteal sciatic nerve block.

### UGNB-Associated Improvement in Pain Scores

For the secondary outcome, Table 3 categorizes patients based on the percentage of pain reduction achieved, ranging from 0% to 25% to 76% to 100%, and provides corresponding frequencies. Among the 1864 of 2735 patients (68.2%) with documented pain scores, a total of 1320 (70.8%) experienced 51% to 100% pain relief following UGNB procedures. Notably, 211 of UGNBs (11.3%) had limited or no pain change after the procedure.

**Table 3.** Distribution of Percent Pain Reduction Among Patients Who Underwent Nerve Block Procedures

Pain reduction distribution, %	Total patients, No. (%) (N = 2735)
Total patients with data	1864 (68.2)
0-25	211 (11.3)
26-50	333 (17.9)
51-75	402 (21.6)
76-100	918 (49.2)

## Discussion

In this study, which to our knowledge is the first to use the multicenter NURVE Block Registry, we found that UGNBs performed in the ED setting were associated with low complication rates and improvement in patient pain scores. Our data support the premise that UGNBs can be incorporated safely into a multimodal regimen for pain control in an acute setting.

In total, 1 major and 9 minor complications were reported in the NURVE Block Registry, representing a complication rate of 0.4%. No patients had significant long-term sequelae from UGNB-associated complications, and no cases of PNI were noted. Cases of PNI, which typically present 48 to 72 hours after procedures, could be missed if the patient did not return to the same site or hospital (or electronically linked hospital center). These data correlate with safety trends reported in anesthesia literature, in which complication rates have also been reported as extremely low in 2 registries and 2 large prospective datasets.^33,34,35,36^ In these studies, the risk of PNI was found to be as low as 0.03%, and local anesthetic toxicity occurred in under 0.02% of cases.^30,33,34,35,36^ As the NURVE Block Registry data increase, updates in complication rates, including delayed complications, will need to be monitored. A future prospective study evaluating PNI will be needed to confirm this, given the inherent limitations of this complication in retrospective review and lack of complete follow-up of all patients at 30 days after a procedure.

The majority of the complications were associated with postprocedural paresthesias and/or weakness. Each case was self-resolving after a brief period (eg, known possible direct effects of the UGNB itself) and thus did not represent true cases of PNI. Clinicians performing UGNBs should be aware and counsel patients on the expected motor and sensory deficits that commonly occur after UGNBs to avoid miscommunications about outcomes and complications. The treating physician should also have a clear plan in place for patients who experience persistent neurological deficits beyond 48 hours. In this study, a transgluteal sciatic nerve block accounted for many of the complications. It is a known risk that patients may get a foot drop associated with the block. Informing the patient of this common sequela (with clear shared decision-making) with use of crutches may prevent falls when motor involvement of the sciatic nerve block occurs.

Regarding efficacy, the NURVE Block Registry demonstrated that 70.8% of UGNBs were considered successful by reducing pain by 51% to 100%. Conversely, 11.3% were considered a failure due to minimal or no pain reduction, which is similar to values cited in anesthesia literature at approximately 5% to 10%, although in these studies, a failed block was typically defined as requiring additional analgesia or general anesthesia.^37,38,39,40,41^ We hypothesize that failure rates may have been, in part, due to operator inexperience. As educational UGNB training programs targeting emergency clinicians grow, we hope that the incidence of failure will decline. Future detailed studies may help to determine the root cause of block failures.

The most common UGNB in the registry was the fascia iliaca block (35.6%), which is typically performed for the indication of acute hip fractures. This block is supported by its extensive literature endorsing the fascia iliaca block utilization in ED settings, as it has been shown to decrease opioid use, enhance pain relief, and lower delirium among the older patient population.^42,43^ The overwhelmingly supportive literature on the efficacy of the fascia iliaca block has translated to the development of standardized workflows that include UGNBs by ED clinicians for hip fractures. Similarly, we believe that the safety data from the NURVE Block Registry for other painful ED conditions (eg, rib fractures, acute sciatica) will encourage clinicians, departments, and hospitals to incorporate UGNBs into the acute pain management protocols and reduce the reliance on opioids to improve patient care.

As accomplished for ED-based airway management by the National Emergency Airway Registry,^44,45^ we recognize the need for a more comprehensive analysis of national trends in ED-based UGNBs’ procedural practice and outcomes. Both the National Emergency Airway Registry and the NURVE Block Registry address ED-based procedures that have the potential to significantly improve patient care and outcomes but also to require specialized procedural competency and training. Despite our data supporting favorable safety and efficacy profiles, nationwide adoption of ED-based UGNB is lacking. Standardized training for emergency medicine clinicians has been cited as a barrier and concern.^46,47,48,49^ Recent data detailing educational initiatives and program development initiatives have been published, alongside guidelines for credentialing emergency physicians.^50,51^ Standardized training will help enable UGNBs to become standards of care in ED-based pain management, which is vital to reducing reliance on opioid monotherapy and offering optimal pain control to patients. As the NURVE Block Registry continues to expand and accumulate data, it will emerge as a vital tool for refining clinical practices and informing policy decisions.

### Limitations

This study has some limitations. As an observational retrospective study, this work is susceptible to potential reporting bias. Moreover, additional bias may be present due to missing data points inherent to retrospective reporting. Differences in retrospective and prospective registry design may have positively or negatively influenced specific variables. A future prospective registry will help confirm our conclusions. While many EDs use a visual analog scale for pain assessment, the lack of formal standardization may hinder comparability across sites. Additionally, most sites involved in the study were academic centers, which could limit the generalizability of the findings. Including a broader diversity of sites is necessary to capture true national trends, as 1 of the sites in this registry performed a large portion of the blocks, potentially making our dataset less generalizable. Another limitation is the reliance on EMRs to capture delayed complications, as some complications may be missed in the medical record review. If patients sought care at an outside facility without data sharing, it is possible that these specific complications were not captured. Therefore, while we acknowledge the presence of delayed complications as a potential issue, readers should consider these findings as indicative rather than definitive, with the understanding that more robust, prospective studies would be necessary to accurately capture and assess these outcomes. Despite these limitations, the substantial number of UGNBs included in this registry makes it less likely that these limitations were associated with our results. To improve data quality in future collections, strategies for data optimization and standardization should be implemented.

## Conclusions

In this cohort study of 2735 UGNB encounters, which used a national UGNB registry, we found that UGNBs performed by ED clinicians were associated with overall improvement in patient pain scores. Data from this registry support the scaling of UGNB training and performance across EDs nationally. The findings advocate the widespread adoption of UGNB training and practice across acute care settings, offering a promising avenue for multimodal analgesia strategies that potentially would reduce opioid use and improve patient care. A large prospective database of ED UGNB will be essential to confirm these findings.

## Notes

Supplement 1. **eTable.** Demographic Data of All 11 Sites

Supplement 2.
Data Sharing Statement
