# Error in Affiliations

**DOI:** 10.1001/jamanetworkopen.2024.55847

**Published:** 2024-12-17

In the Original Investigation titled “Complication Rates After Ultrasonography-Guided Nerve Blocks Performed in the Emergency Department,”^1^ published November 13, 2024, the affiliation for Drs Martin and Nagdev was incorrect; both authors are affiliated with the Department of Emergency Medicine, Wilma Chan Highland Hospital Campus, Alameda Health System, University of California, San Francisco. This article has been corrected.
