# Supplementary material for: Complication Rates After Ultrasonography-Guided Nerve Blocks Performed in the Emergency Department
Source: JAMA Netw Open. 2024 Nov 13;7(11):e2444742. doi: 10.1001/jamanetworkopen.2024.44742 (PMC11561692; doi:10.1001/jamanetworkopen.2024.44742)
Supplement: Supplement 1. — eTable. Demographic Data of All 11 Sites [file jamanetwopen-e2444742-s001.pdf]

Supplemental Online Content

Goldsmith A, Driver L, Duggan NM, et al. Complication rates after ultrasonography-guided nerve blocks performed in the emergency department. *JAMA Netw Open*. 2024;7(11):e2444742. doi:10.1001/jamanetworkopen.2024.44742

eTable. Demographic Data of All 11 Sites

This supplemental material has been provided by the authors to give readers additional information about their work.

© 2024 Goldsmith A et al. *JAMA Network Open*.

eTable. Demographic Data of All 11 Sites

Site number	Number of blocks in registry	Annual patient volume	Urban/Rural/Community (or anything else specific to location)	Residency?	Length of Residency	Fellowship?	Number US faculty	Level of Trauma Center
1	157	110000	urban	Yes	4	No	6	1
2	268	120000	urban	yes	4	yes	4	1
3	145	100000	urban	yes	3	yes	6	1
4	973	70000	urban	yes	4	yes	7	1
5	360	110000	urban	yes	4	yes	6	1
6	147	78000	Urban	Yes	4	Yes	7	1
7	180	65000	urban	yes	4	yes	4	1
8	52	35000	urban	no			3	3
9	113	130000	Urban	Yes	4	Yes	10	1
10	288	45000	Community	yes	3	yes	6	2
11	52	80000	Urban	Yes	3	Yes	6	1

© 2024 Goldsmith A et al. *JAMA Network Open*.
