# Supplementary material for: Complication Rates After Ultrasonography-Guided Nerve Blocks Performed in the Emergency Department
Source: JAMA Netw Open. 2024 Nov 13;7(11):e2444742. doi: 10.1001/jamanetworkopen.2024.44742 (PMC11561692; doi:10.1001/jamanetworkopen.2024.44742)
Supplement: Supplement 2. — Data Sharing Statement [file jamanetwopen-e2444742-s002.pdf]

Data Sharing Statement

Goldsmith. Complication Rates After Ultrasonography-Guided Nerve Blocks Performed in the Emergency Department. *JAMA Netw Open*. Published November 13, 2024. doi:10.1001/jamanetworkopen.2024.44742

Data
Data available: No

Additional Information
Explanation for why data not available: Did not consent patients for public data
